# Increased Levels of HbA1c in Individuals with Type 2 Diabetes and Depression: A Meta-Analysis of 34 Studies with 68,398 Participants

**DOI:** 10.3390/biomedicines10081919

**Published:** 2022-08-08

**Authors:** Alma Delia Genis-Mendoza, Thelma Beatriz González-Castro, Gisselle Tovilla-Vidal, Isela Esther Juárez-Rojop, Rosa Giannina Castillo-Avila, María Lilia López-Narváez, Carlos Alfonso Tovilla-Zárate, Juan Pablo Sánchez-de la Cruz, Ana Fresán, Humberto Nicolini

**Affiliations:** 1 Laboratorio de Genómica de Enfermedades Psiquiátricas y Neurodegenerativas, Instituto Nacional de Medicina Genómica, Ciudad de México 14610, Mexico; 2 División Académica Multidisciplinaria de Jalpa de Méndez, Universidad Juárez Autónoma de Tabasco, Jalpa de Méndez 86040, Tabasco, Mexico; 3 División Académica de Ciencias de la Salud, Universidad Juárez Autónoma de Tabasco, Villahermosa 86100, Tabasco, Mexico; 4 Hospital Chiapas Nos Une “Dr. Gilberto Gómez Maza”, Secretaría de Salud de Chiapas, Tuxtla Gutiérrez 29045, Chiapas, Mexico; 5 División Académica Multidisciplinaria de Comalcalco, Universidad Juárez Autónoma de Tabasco, Comalcalco 86040, Tabasco, Mexico; 6 Subdirección de Investigaciones Clínicas, Instituto Nacional de Psiquiatría Ramón de la Fuente Muñíz, Ciudad de México 14370, Mexico

**Keywords:** diabetes, HbA1c, depression, hypoglycemic drug, complications

## Abstract

Glycosylated hemoglobin is used to diagnose type 2 diabetes mellitus and assess metabolic control. Depression itself has been associated with high levels of HbA1c in individuals with T2DM. The association between diabetes and depression suggests the usefulness of determining HbA1c as a biological marker of depressive symptoms. The aim of this study was to determine HbA1c levels in individuals with T2DM with vs. without depression. Additionally, we analyzed the influence of pharmacological treatments, time of evolution, and complications of disease. We performed a literature search in different databases published up to January 2020. A total of 34 articles were included. Our results showed that individuals with T2DM with depression showed increased levels of HbA1c in comparison to individuals with T2DM without depression (d = 0.18, 95% CI: 0.12–0.29, p(Z) < 0.001; I^2^ = 85.00). We also found that HbA1c levels remained elevated in individuals with T2DM with depression who were taking hypoglycemic drugs (d = 0.20 95% CI: 0.11–0.30, p(Z) < 0.001; I^2^ = 86.80), in individuals with less than 10 years of evolution (d = 0.17 95% CI: 0.09–0.26, p(Z) = 0.001; I^2^ = 66.03) and in individuals with complications of the disease (d = 0.17, 95% CI: 0.07–0.26, p(Z) < 0.001; I^2^ = 58.41). Our results show that HbA1c levels in individuals with T2DM with depression are significantly increased compared to controls with T2DM without depression. Additionally, these levels remained elevated in individuals who were taking hypoglycemic drugs, those with less than 10 years of disease evolution, and those with complications related to diabetes. It is necessary to examine the existence of a diabetes–HbA1c–depression connection.

## 1. Introduction

Type 2 diabetes mellitus (T2DM) is a chronic metabolic disease. Globally, the prevalence of type 2 diabetes mellitus is already high, and it is increasing everywhere in the world, including Mexico. Mexico ranks sixth worldwide, with 12.8 million people with diabetes. Furthermore, diabetes is among the leading causes of death globally and regionally, mainly due to its serious complications [1,2].

In recent years, various studies have shown that individuals with T2DM have a doubled risk of depression compared to individuals without T2DM; in fact, there is a two-way association between depression and T2DM, and each one increases the risk of the other [3,4]. Depressive symptoms in individuals with T2DM are associated with poor glycemic control, impaired physical functioning, low quality of life, hospitalizations, diabetic complications, and high rates of mortality. However, depression is poorly recognized in T2DM and it is usually untreated [5]. 

According to the American Diabetes Association, hemoglobin A1c (HbA1c) can be used as a diagnostic test for diabetes and as a test for defining glycemic control in people with diabetes mellitus [6]. In fact, there is evidence of the utility of HbA1c as a useful predictor of diabetes risk, and it can be used to identify pre-diabetes with other type 2 diabetes risk factors [7]. As depression and diabetes are reciprocally linked [8], after using HbA1c as proof of the glycemic condition in T2DM, an inverse correlation could be used between depressive symptoms and HbA1c levels [9]. Therefore, it is plausible to assume that there is a diabetes–HbA1c–depression connection. This relationship has been linked to negative moods [10] and a greater risk of diabetes complications in general. Nonetheless, no conclusive results have been reached, due to the lack of studies performed. The aim of the present study was to compare the HbA1c levels in patients with T2DM with and without depression. In addition, we performed a series of meta-analyses to examine potential moderators: first, according to the pharmacological treatment; second, longitudinally, according to the time of evolution of diabetes; and third, according to the T2DM complications.

## 2. Materials and Methods

This study was designed as a systematic review and meta-analysis to evaluate the possible participation of HbA1c levels in individuals with type 2 diabetes mellitus and depression. The protocol was registered at the International Prospective Register of Systematic Reviews (PROSPERO registration number: CRD42021282479).

### 2.1. Search Strategy

This meta-analysis review followed the strategy of the PRISMA statement. We performed a literature search in Embase, PubMed, Cochrane, Web of Knowledge, FDA.gov, and ClinicalTrials.gov databases. An initial search was performed using the keywords: “HbA1c”, “Depression”, “Diabetes”, “Diabetes Mellitus”, “hemoglobin A1c”, and “glycated hemoglobin”. The search also included medical subject terms (MeSH): (HbA1c) AND “Depression” (Mesh). (hemoglobin A1c) AND “Depression” (Mesh), (glycated hemoglobin) AND “Depression” (Mesh), (HbA1c) AND “Diabetes Mellitus” (Mesh), (hemoglobin A1c) AND “Diabetes Mellitus” (Mesh), (glycated hemoglobin) AND “Diabetes Mellitus” (Mesh), and publication types based on the PICO framework (participants, comparison, intervention, and outcomes). 

A reference list of papers, as well as the latest editions of relevant journals not available online, were scrutinized for new references. When the information was not clear, the full text was obtained to review it. The corresponding authors of potentially eligible studies were contacted when their study reported data impossible to discriminate. We considered all potentially eligible studies for review.

### 2.2. Study Selection

The inclusion criteria were (1) only articles published in English, (2) studies that measured HbA1c levels in plasma, (3) association studies between cases (individuals with T2DM and depression) and controls (individuals with T2DM without depression), (4) studies that performed the same evaluations in cases and controls (Mean (SD)). To sum up, the studies were selected regardless of the methods used, if they reported a baseline outcome measure of diabetes within an adult population (≥ 18 years) with type 2 diabetes. Measurements of glycosylated hemoglobin A1c (HbA1c) value and depression had to be obtained with validated scales. To ensure external validity, experimental studies were included when the samples adopted broad inclusion criteria (type 2 diabetes, HbA1c, and depression).

The exclusion criteria were (1) studies that included individuals with type 1 diabetes, (2) duplicated studies, (3) studies that did not measure HbA1c concentrations, (4) studies that did not show standard deviation or mean of levels and did not provide enough information to calculate them.

### 2.3. Data Extraction

Data extracted for this systematic review and meta-analysis included: year of publication, location, the scale for the diagnosis of depression, evolution duration of T2DM in years, presence of T2DM complications, hypoglycemic treatment, sample size, units of measurement, mean, and standard deviation of the mean. One of the selected studies [11] applied two scales to the same study population. However, the results were different for each scale; therefore, we considered it twice. Two reviewers independently read and extracted all the information to prevent potential errors. Disagreements about the inclusion and exclusion criteria were solved by discussion; a third reviewer adjudicated any disputes. 

### 2.4. Quality Score Assessment

The methodological quality was assessed using the Newcastle-Ottawa Assessment Scale (NOS) [12]. The cut-off point of the studies included was determined with scores of six or higher.

### 2.5. Statistical Analysis

Data from the studies were extracted into a spreadsheet. We used the “d” statistic and 95% confidence interval (95% CI) to estimate the mean differences in HbA1c levels in individuals with T2DM and depression compared to individuals with T2DM without depression. Considering that HbA1c levels are related to multiple variables and may vary from one observation to another, the pooled weighted mean differences and 95% CI were calculated using the DerSimonian and Laird random model. The heterogeneity among studies was evaluated using the Cochran Q test and inconsistency index (I^2^). We considered a *P* value of <0.10 as significant and indicative of heterogeneity. We also calculated the I^2^ metric, values <25% were considered as absent of heterogeneity, values between 25 and 50% as moderate heterogeneity, while values >75% were indicative of high heterogeneity. To explore the robustness of the results (e.g., in HbA1c measurement methods, the sample size, the quality of heterogeneity) sensitivity analyses were performed by excluding specific studies.

Five between-group meta-analyses of the HbA1c levels in individuals with T2DM and depression were performed: (1) T2DM and depression, compared to T2DM without depression; (2) individuals with T2DM and depression who were taking hypoglycemic drugs, compared to T2DM without depression and who were taking hypoglycemic drugs; (3) T2DM and depression with less than 10 years of disease evolution, compared to T2DM without depression and less than 10 years of disease evolution; (4) T2DM and depression with more than 10 years of evolution, compared to T2DM without depression and more than 10 years of evolution, and (5) depression and T2DM with complications compared to non-depressed patients with T2DM and complications (e.g., hyperlipidemia or retinopathy).

## 3. Results

### 3.1. Study Information

**Figure 1:**
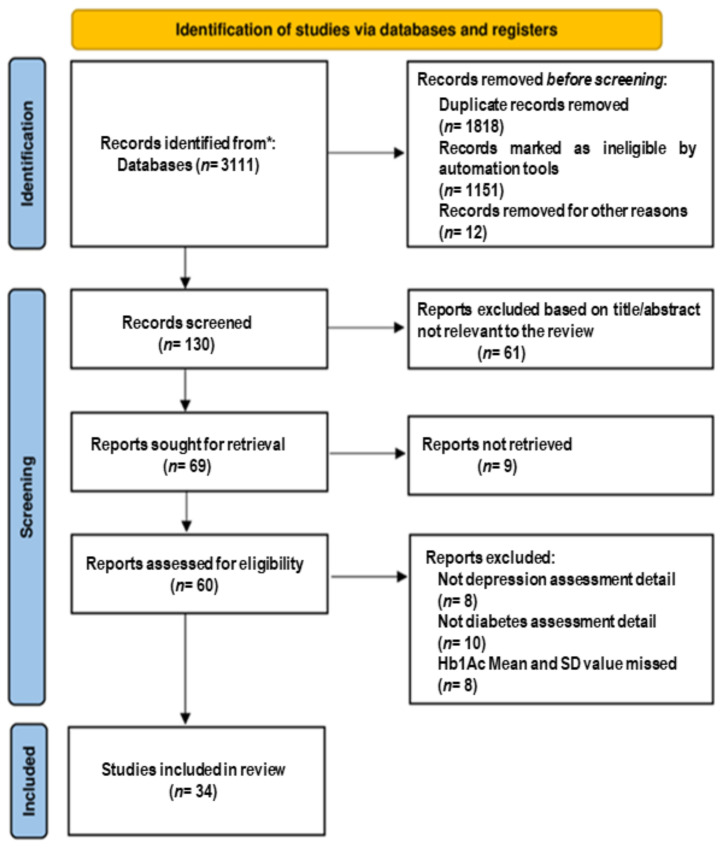
Flowchart showing the different phases in the systematic review and meta-analysis.

After a detailed evaluation, 34 studies were chosen for having the necessary data to be included in this meta-analysis [4,11,13,14,15,16,17,18,19,20,21,22,23,24,25,26,27,28,29,30,31,32,33,34,35,36,37,38,39,40,41,42,43]. A detailed chart of all the studies found was created, where we specified the reasons for inclusion or rejection. The process of the study selection is shown in Figure 1. The characteristics of the studies included are summarized in Table 1. This meta-analysis included 6094 individuals with T2DM and depression and 62,304 individuals with T2DM without depression as the comparison group. The quality assessment is represented in Table 2. 

### 3.2. Meta-Analysis of Individuals with T2DM with and without Depression

**Figure 2:**
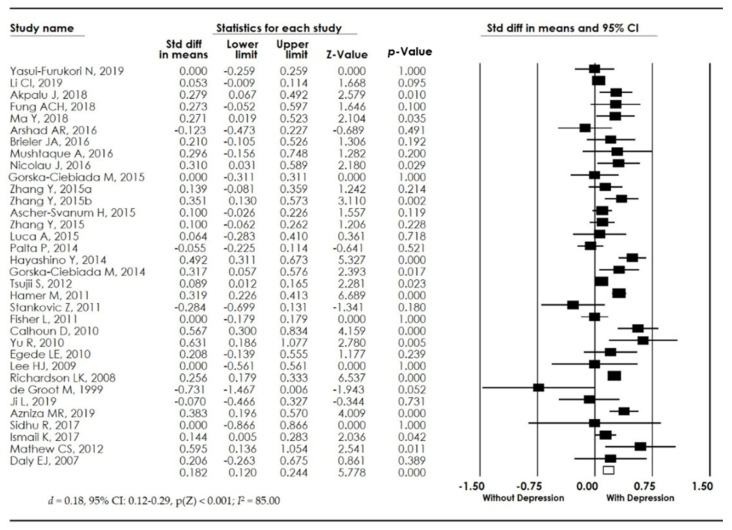
Forest plot of the meta-analysis for HbA1c levels in individuals with type 2 diabetes mellitus (T2DM) and depression versus individuals with type 2 diabetes mellitus without depression [4,11,13,14,15,16,17,18,19,20,21,22,23,24,25,26,27,28,29,30,31,32,33,34,35,36,37,38,39,40,41,42,43].

First, we analyzed the total sample (*n* = 68,398), of which 6094 individuals had T2DM and depression and 62,304 had T2DM without depression. We observed that individuals with T2DM and depression showed higher levels of HbA1c in comparison to those without depression (*d* = 0.18, 95% CI: 0.12-0.29, p(Z) < 0.001; I^2^ = 85.00). Our results suggest that in the presence of depression, the levels of HbA1c increase in individuals with T2DM (see Figure 2).

### 3.3. Meta-Analysis of T2DM with/without Depression When Using Hypoglycemic Drugs

**Figure 3:**
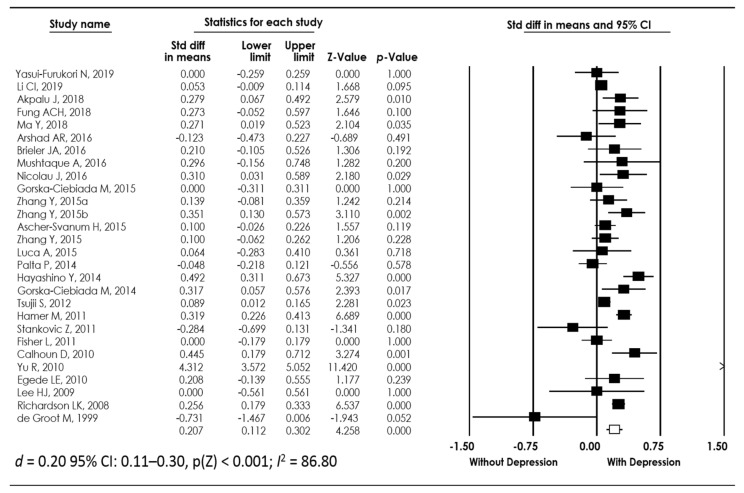
Forest plot of the meta-analysis for HbA1c levels in individuals with type 2 diabetes mellitus with/without depression when using hypoglycemic drugs [4,11,13,16,17,18,19,22,23,24,25,26,27,28,29,30,31,32,33,35,36,37,38,39,40,41,43].

We wanted to know if the HbA1c levels were higher in individuals with T2DM and depression who were taking hypoglycemic drugs compared to T2DM individuals without depression who were also taking hypoglycemic drugs. In this sub-group meta-analysis, the sum of samples consisted of 28 studies [4,11,13,16,17,18,19,22,23,24,25,26,27,28,29,30,31,32,33,35,36,37,38,39,40,41,43], providing data on 65,864 individuals, of whom 5566 had T2DM and depression and were taking hypoglycemic drugs, while 60,298 had T2DM without depression and were also taking hypoglycemic drugs. This analysis showed that individuals with T2DM who were treated with hypoglycemic drugs and presented depression showed increased levels of HbA1c (*d* = 0.20 95% CI: 0.11–0.30, p(Z) < 0.001; I^2^ = 86.80), which means that HbA1c levels remained high in individuals with T2DM and depression, even when they were taking hypoglycemic drugs; Figure 3 and Table 3. 

### 3.4. Meta-Analysis of T2DM with/without Depression Depending on the Duration of Evolution

**Figure 4:**
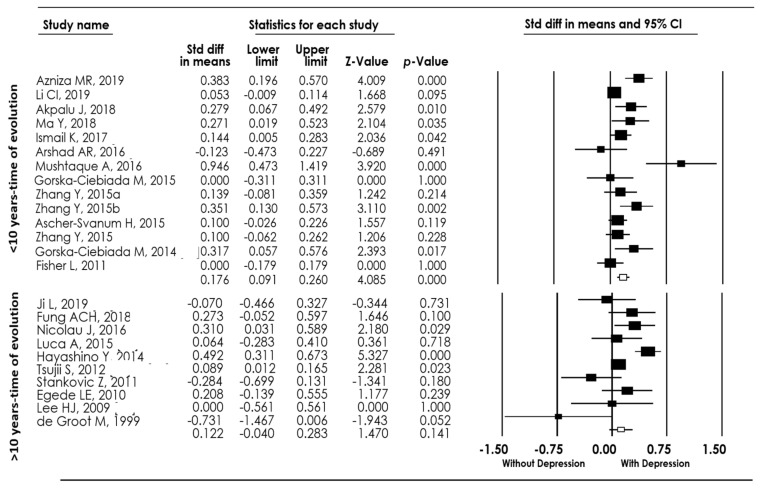
Forest plot of the meta-analysis for HbA1c levels in individuals with type 2 diabetes mellitus (T2DM) and with/without depression regarding the duration of diabetes evolution [4,11,14,15,16,17,18,19,21,22,24,25,26,27,28,29,28,29,30,31,32,33,36,37,40,43].

We performed a subgroup analysis according to the duration of the T2DM evolution. Fourteen studies reported individuals with less than ten years of evolution [11,15,16,17,19,21,22,24,26,27,28,32,37]. Then, we wanted to know if HbA1c levels were higher in individuals with less than 10 years of T2DM evolution and depression, compared to individuals with less than 10 years of T2DM evolution but without depression. We found that individuals with T2DM and depression with less than 10 years of evolution showed increased levels of HbA1c (*d* = 0.17 95% CI: 0.09–0.26, p(Z) = 0.001; I^2^ = 66.03) Figure 4. On the other hand, ten studies reported that individuals with more than ten years of T2DM evolution [4,14,18,25,29,31,33,36,40,43] and we found no statistical differences in the mean concentration of HbA1c when compared individuals with depression versus those without depression (*d* = 0.12 95% CI: −0.40–0.28, p(Z) = 0.14; I^2^ = 69.33). 

### 3.5. Meta-Analysis of Complications of T2DM with/without Depression

**Figure 5:**
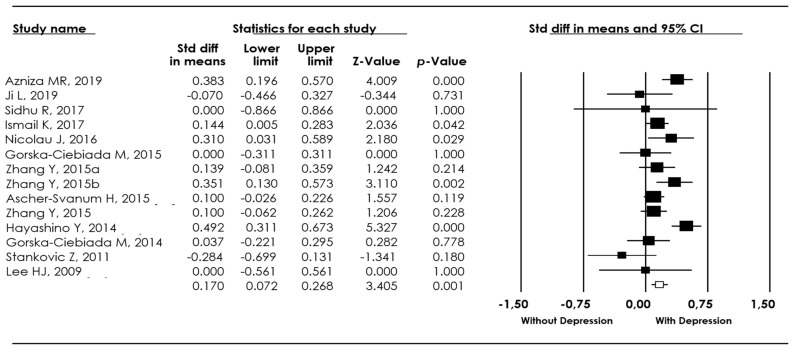
Forest plot of the meta-analysis for HbA1c levels in individuals with/without type 2 diabetes mellitus (T2DM)-related complication analysis [11,14,15,20,21,25,26,27,28,31,32,36,40].

Finally, we analyzed individuals with T2DM with/without depression who presented complications due to diabetes. We wanted to know if having depression and complications had any influence on the levels of HbA1c. We evaluated a total of 11,344 individuals, of whom 1695 presented T2DM with complications and depression. Fourteen studies were included in this meta-analysis [11,14,15,20,21,25,26,27,28,31,32,36,40]. We found statistically significant high levels of HbA1c in T2DM patients with complications and depression compared to non-depressed individuals with T2DM and complications (*d* = 0.17, 95% CI: 0.07–0.26, p(Z) < 0.001; I^2^ = 58.41) Figure 5. 

## 4. Discussion

The main challenge for people living with T2DM is to have optimal glycemic control in order to minimize the risk of life-changing complications. In this sense, HbA1c is an important indicator of metabolic control and diabetes management. On the other hand, there is a multi-directional relation between metabolic control and depression (or other mood disorders) in individuals with T2DM. For that reason, our primary aim was to evaluate the link between type 2 diabetes mellitus, depression, and levels of HbA1c. Additionally, we addressed the influence of moderators, such as the pharmacological treatment, duration of evolution, and diabetes complications, in this triad (type 2 diabetes mellitus-depression-HbA1c levels).

We started by addressing the relation between depression in individuals with T2DM and the concentrations of HbA1c. We found that these individuals presented higher levels of HbA1c than those with T2DM but without depression. This outcome indicates that there is a link between depression and glycemic control. To date, elevated levels of HbA1c in patients with diabetes are not considered biomarkers for depression in patients with T2DM. Nonetheless, the present meta-analysis indicates that high HbA1c levels may be a good indicator for clinicians to explore the presence of depression in individuals with T2DM. Detecting symptoms of depression could be very useful in medical practice, as people living with depressive symptoms have emotional difficulties that could interfere with getting used to having diabetes and following health advice [44,45]. Additionally, individuals with T2DM and depression may have high levels of stress daily, which could lead to difficulties in manning an appropriate diet, adequate exercise, and adherence to treatment among other daily activities that are necessary to have good glycemic control [46]. 

Individuals with T2DM complications were divided into with and without depression. We observed that those with T2DM complications and depression showed higher levels of HbA1c than those without T2DM complications [46,47]. This is similar to previous findings; depression is directly associated with poor diet adherence, which could result in poor metabolic control leading to long-term complications [48,49]. Then, our outcomes support the importance of the use of HbA1c levels for early detection of depression, as well as the reduction of depressive symptoms in individuals with T2DM in order to control the disease. Despite our findings, we cannot suggest the use of the HbA1c biomarker solely as a diagnostic criterion for depression, but rather as a predictor of depressive symptoms and metabolic control in patients with diabetes mellitus. Identifying depressive symptoms early helps establish antidepressant treatment and achieve metabolic control [23]. Currently, biomarkers have been studied in the diagnosis of depressive disorder [50]; however, we do not have specific biomarkers. In patients with diabetes mellitus, determining HbA1c and investigating depressive symptoms can be of clinical utility.

To better understand the participation of HbA1c in depression in individuals with T2DM, we analyzed the duration of the evolution of the disease. Individuals with T2DM and depression who had less than 10 years of diabetes evolution showed increased levels of HbA1c. Besides the impact that depression could have on the life of individuals with T2DM, our results indicated that HbA1c and depressive symptoms have a bigger effect in those individuals with fewer years of diabetes evolution. On the other hand, when we analyzed the HbA1c in patients with T2DM with more than 10 years of the disease, no differences were observed. This suggests that in patients with T2DM of more than 10 years of disease, the concentrations of HbA1c should not be used as a biomarker for depression. In addition to negative coping, the presence of depressive symptoms and the combination of factors will probably have a significant negative effect on individuals with T2DM [51,52]. Therefore, we recommend early screening for psychological comorbidities in individuals with T2DM; particularly during the first 10-year onset of the disease. 

### Meta-Analysis of T2DM with/without Depression When Using Hypoglycemic Drugs

Finally, in order to better understand how the triad T2DM–depression–HbA1c is linked, we analyzed the relationship between individuals with T2DM with/without depression when using hypoglycemic drugs. This analysis showed that those individuals with depression had higher levels of HbA1c levels in comparison to individuals with T2DM without depression who were under hypoglycemic drug treatment. This is similar to some studies indicating that despite being adherent to their medications, a considerable percentage of individuals with T2DM have poor glycemic control [53]. The reasons could vary widely; explanations focus on the accessibility to medical care (the severity of disease, dosage prescribed, and overall efficacy of the chosen medication). Therefore, there should be better decisions on drug treatment, particularly at the beginning of diagnosing T2DM and behavior disorders [54,55]. Finally, as the adherence to treatment using hypoglycemic medication did not regulate the levels of HbA1c, it is necessary to emphasize the management of depressive symptoms in order to improve glycemic control among individuals with type 2 diabetes mellitus. Moreover, it is necessary to consider a new class of drugs for T2DM, such as glucagon-like peptide 1 agonists that seem to have multiple actions in the central nervous system, including antidepressants that can affect the control of diabetes and depression.

Nowadays, it is well known that in the brain, insulin performs many activities, such as promoting neuronal growth, preventing apoptosis, and reducing inflammation; moreover, it is related to serotonin levels and the activity of monoamine oxidase [56]. Moreover, selective serotonin reuptake inhibitor (SSRI) medications are widely used and accepted for depressive disorders. Hence, there is evidence that shows increased insulin sensitivity and altered HbA1c levels as consequences of SSRI medication, such as fluoxetine [57]. In fact, the long-term safety of these medications is associated with a higher risk of diabetes [56]. For that reason, it is necessary to consider the antidepressant medications of the patients.

We want to highlight some limitations when interpreting our results. It is well known that depression could derive from multifactorial causes, including income, education, and access to private health, among others; these factors could increase the risk of developing depression in individuals with T2DM. Therefore, one limitation of our study is that we did not evaluate these data due to the lack of information in the studies included. Furthermore, it is important to consider the stigma attached to a mental disorder diagnosis, and sometimes it is difficult for individuals with T2DM to request or accept a psychiatric evaluation. Although the diagnosis of depressive symptoms as a screening result is helpful, the diagnosis of depression should be performed through international criteria (DSM-V, ICD-10). As not all the studies we included specified the methodological diagnosis of depression, this could also be considered a limitation. We could not establish the chronicity and the physiological impact of the diagnosis of diabetes; these factors may have had a significant moderator role. Another limitation was regarding the hypoglycemic drug analysis, a considerable percentage of studies measured adherence from administrative data, so we cannot know for sure if the participants actually took their medication. We could not analyze confounding factors that influence glycemic control, such as diet and exercise. Furthermore, the sub-analysis comparing individuals with 10 years of T2DM evolution or more has an important limitation in the measurements of the effect. The eligible articles are represented in the means, so the findings should be interpreted with considerable caution. Therefore, the findings could have skewed the outcomes in the wrong direction. Regardless of these limitations, we want to emphasize the important HbA1c levels as possible predictors of the dangerous interaction between T2DM and depression. Moreover, it is necessary to consider that depression unfolds a sustained form of psychosocial stress, which induces patterns of psycho-biological consequences that may lead to a distorted metabolism.

## 5. Conclusions

In conclusion, our meta-analysis of 34 studies comprising 68,398 individuals with T2DM revealed that HbA1c levels are increased in individuals with type 2 diabetes mellitus and depression; this is also observed in individuals with T2DM and depression who present diabetic complications, those with less than 10 years of evolution, and in those using hypoglycemic drugs. Therefore, our findings suggest that people diagnosed with T2DM and depressive symptoms have high levels of HbA1c; however, the biomarker should not be used individually as a diagnostic criterion for depression. Its usefulness in medical practice is that clinicians should explore depressive symptoms in patients with T2DM with elevated HbA1c. Identifying depressive symptoms early helps establish antidepressant treatment and achieve metabolic control. At the same time, we suggest an early psychiatric evaluation in individuals with T2DM and a good follow-up in order to prevent complications of diabetes. 

## Figures and Tables

**Table 1:** Characteristics of the studies included in this meta-analysis.

Information about the Study							Clinical Information		
First Author	Location	Year	Total Sample	T2DM and Depression	T2DM without Depression	Assessment of Depression	Time since Diagnosis (in Years)	Comorbidities	Complications
Yasui-Furukori [13]	Hirosaki	2019	435	68	367	CES-D	N/A	N/A	N/A
Ji L [14]	China	2019	162	30	132	PHQ-9	10.5 ± 8.0	N/A	89.5% Diabetic peripheral neuropathy
Azniza MR [15]	Malaysia	2019	511	164	347	M-GDS-14	8.57±5.57	44% Comorbidities	75% Diabetic complication
Li CI [16]	Taiwan	2019	32,829	1041	31,788	ICD-9-CM	7.23	36.4% Obesity, 4.88% stroke, 8.66% CAD, 2.47% CHF, 2.03% cancer, 25.86% hyperlipidemia, 45.40% HA, 0.49% atrial fibrillation, 9.69% chronic hepatitis, 4.45% COPD, 0.38% hypoglycemic	N/A
Akpalu J [17]	Ghana	2018	400	125	275	PHQ-9	9.1 ± 7.3	47.2% Obesity, 79.5% HA	N/A
Fung ACH [18]	Hong Kong	2018	325	42	283	GDS-15	12.0±8.3	29% CAD, stroke, 52% CVC, chronic kidney disease, and any form of cancer	N/A
Ma Y [19]	Beijing	2018	245	114	131	ATQ-30, SDS	9.33±1.9	N/A	N/A
Sidhu R [20]	Canada	2017	41	6	35	PHQ-9	N/A	N/A	51.2% Distress
Ismail K [21]	London	2017	1651	232	1419	PHQ-9	N/A	N/A	1.45% MI, 0.72% stroke, 0.78% incident carotid/limb revascularization or amputation, 2.42% incident macrovascular, 9.81% retinopathy, 6.8% neuropathy, 9.4% nephropathy, 23.5% microvascular complications
Arshad AR [22]	Pakistan	2016	133	51	82	PHQ-9	3	48.87% HA	N/A
Brieler JA [23]	USA	2016	1174	40	1134	ICD-9-CM codes	N/A	4.9% anxiety disorder, 74.1% obesity, 65.8% hyperlipidemia, 83.3% HA, 33.9% CVD	N/A
Mushtaque A [24]	India	2016	80	31	49	HAM-D	4.58±2.12	N/A	N/A
Nicolau J [25]	Spain	2016	200	100	100	BDI	12.81±10.24	N/A	69.5% HA, 49% dyslipidemia, 16.5% CAD, 5.5% stroke, 46% obesity, 5% vasculopathy, 4.5% chronic kidney disease, 4% CHF, 7.5% nephropathy, 9% retinopathy, 10.5% neuropathy, 3% diabetic foot
Gorska-Ciebiada [26]	Poland	2015	189	57	132	GDS-30	8.69 ± 6.23	13.4% Lung disease, 20.6% atrial fibrillation, 21% CHF, 39.8% gastrointestinal tract disease, 21.7% kidney disease, 26.8% thyroid disease, 39.5% CVD	78.9% Hyperlipidemia, 43.8% retinopathy, 35.1% nephropathy, 20.2% neuropathy, 42.3% hypoglycemia
Ascher-Svanum H [27]	Europe	2015	971	485	486	EuroQol-5D	9.96±7.01	N/A	31.4% Macrovascular, 37.7% microvascular
Zhang Y [11]	China	2015	545	96	449	PHQ-9	6.0±3.0	78.9% HA, 85.3% dyslipidemia, 30.5 albuminuria	9.7% CVD, 20.6% retinopathy, 1.8% chronic kidney disease, 2.4% sensory neuropathy
Zhang Y [11]	China	2015	545	97	449	CES-D	6.0±3.0	78.9% HA, 85.3% dyslipidemia, 30.5 albuminuria	9.7% CVD, 20.6% retinopathy, 1.8% chronic kidney disease, 2.4% sensory neuropathy
Luca A [29]	Italy	2015	128	65	63	HAM-D	11.9±9.9	N/A	N/A
Palta P [30]	USA	2014	564	218	346	Short-CARE	N/A	N/A	N/A
Zhang Y [28]	China	2015	2538	155	2383	PHQ-9	6.0±2.0	78.5% HA, 91.3% dyslipidemia	8.3% CAD, 3.6% stroke, 13.2% sensory neuropathy, 11.9% retinopathy, 15.9% peripheral vascular disease, 2.4% chronic kidney disease, 18.7% microalbuminuria, 5.7% microalbuminuria, 0.4% end-stage renal disease
Hayashino Y [31]	Japan	2014	3573	122	3451	PHQ-9	14.6±10.1	16.5% CVD, 9.8% cancer, 0.22% arthritis	41.9% Retinopathy, 54.4% nephropathy
Gorska-Ciebiada [32]	Poland	2014	276	82	194	GDS-30	8.69 ± 6.23	39.5% CVD, 5.07% Stroke, 77.17% HA, 78.9% hyperlipidemia	43.8% Retinopathy, 35.1% nephropathy, 20.2% neuropathy
Tsujii S [33]	Japan	2012	3305	919	2386	CES-D	13.8 ±9.8	N/A	N/A
Mathew CS [34]	India	2012	80	31	49	MDI, BDI	N/A	N/A	N/A
Hamer M [35]	London	2011	4338	498	3840	CES-D	N/A	N/A	N/A
Stanković Z [36]	Serbia	2011	90	46	44	PHQ, MINI, BDI,	11.96±6.34	N/A	86.6% Neuropathy, 42.2% retinopathy, 21.1% nephropathy
Fisher L [37]	USA	2011	483	256	227	PHQ-8	7.6±6.1	N/A	N/A
Calhoun D [38]	USA	2010	581	61	520	CES-D	N/A	N/A	N/A
Yu R [39]	China	2010	100	28	72	SDS	N/A	N/A	N/A
Egede LE [4]	South Carolina	2010	201	40	161	CES-D	12.5±9.1	N/A	N/A
Lee HJ [40]	Maryland	2009	49	23	26	BDI-II; IDS-SR	11.9±8.49	N/A	29.1% CAD, 78.2% hypertension, 67.3% hyperlipidemia, 65.5% obesity, 12.7% nephropathy, 40% neuropathy, 21.8% retinopathy
Richardson LK [41]	USA	2008	11,525	696	10,829	ICD-9-CM	N/A	6.05% Stroke, 26.6% CHD, 51.85% HA	N/A
Daly EJ [42]	Texas	2007	89	65	24	PHQ-2, QIDS-SR	N/A	N/A	N/A
de Groot M [43]	USA	1999	39	10	29	SCID	11.1±6.35	N/A	N/A

CESD-R: Center Epidemiología studies Depresión scale-Revisen; PHQ-9: patient health questionnaire- 9; CHF: congestive heart failure; CAD: coronary artery disease.

**Table 2:** Quality assessment of the studies included based on the Newcastle-Ottawa scale.

First Author	Selection				Comparability	Exposure			Total
	Adequate Case Definition	Representativeness of the Cases	Selection of Controls	Definition of Controls	Comparability of Cases and Controls	Ascertainment of Exposure	Method of Ascertainment	Non-Response Rate	
Yasui-Furukori [13]	*	*	*	*	*	**	*		8
Ji L [14]	*	*	*	*	**	*	*		8
Azniza MR [15]	*		*	*	*	*	*		6
Li CI [16]	*	*	*	*	*	*	*		7
Akpalu J [17]	*	*	*	*	*	*	*		7
Fung ACH [18]	*	*	*	*	*	*	*		7
Ma Y [19]	*		*	*	*	*	*		6
Sidhu R [20]	*		*	*	*	*	*		6
Ismail K [21]	*	*	*	*	*	**	*		8
Arshad AR [22]	*	*	*	*	*	*	*		7
Brieler JA [23]	*		*	*	*	*	*		6
Mushtaque A [24]	*		*	*	*	*	*		6
Nicolau J [25]	*	*	*	*	*	**	*	*	9
Gorska-Ciebiada [26]	*	*	*	*	*	**	*		8
Ascher-Svanum H [27]	*	*	*	*	**	*	*		8
Zhang Y [11]	*	*	*	*	*	**	*		8
Zhang Y [11]	*	*	*	*	**	*	*		8
Luca A [29]	*	*	*	*	*	*	*		7
Palta P [30]	*		*	*	*	*	*		6
Zhang Y [28]	*	*	*	*	**	*	*		8
Hayashino Y [31]	*	*	*	*	**	*	*		8
Gorska-Ciebiada [32]	*	*	*	*	*	**	*		8
Tsujii S [33]	*		*	*	*	*	*		6
Mathew CS [34]	*		*	*	*	*	*		6
Hamer M [35]	*	*	*	*	*	*	*		7
Stanković Z [36]	*	*	*	*	*	*	*		7
Fisher L [37]	*		*	*	*	*	*		6
Calhoun D [38]	*	*	*	*	*	**	*	*	9
Yu R [39]	*		*	*	*	*	*		6
Egede LE [4]	*		*	*	*	*	*		6
Lee HJ [40]	*		*	*	*	*	*		6
Richardson LK [41]	*		*	*	*	*	*		6
Daly EJ [42]	*		*	*	*	*	*		6
de Groot M [43]	*		*	*	*	*	*		6

* One point, ** Two points.

**Table 3:** Hypoglycemic and depression information of the patients studied.

First Author	Treatment	Antidepressant Information
Yasui-Furukori [13]	64.1% Oral agents; 35.9% insulin	N/A
Ji L [14]	N/A	N/A
Azniza MR [15]	N/A	N/A
Li CI [16]	1.2% No medication; 82.45% oral agents; 2.85% insulin; 13.5% insulin + oral agents	N/A
Akpalu J [17]	N/A	Use of depression medication was part of the exclusion criteria
Fung ACH [18]	7% Lifestyle only, 69% oral agents, 4% insulin, 20% insulin + oral agents	This was cited as a limitation
Ma Y [19]	100% Oral agents	N/A
Sidhu R [20]	N/A	N/A
Ismail K [21]	53.2% Oral agents, 3.2% insulin	N/A
Arshad AR [22]	N/A	Use of depression medication was part of the exclusion criteria
Brieler JA [23]	35% Insulin, 76.9% oral agents.	Tricyclic antidepressants; selective serotonin reuptake inhibitors; serotonin and norepinephrine reuptake inhibitors; and non-classified antidepressants
Mushtaque A [24]	100% Insulin	Use of depression medication was part of the exclusion criteria
Nicolau J [25]	68.8% Oral agents, 14.75% insulin basal, 11.15% biphasic insulin, 5.3% basal bolus regimen	Use of depression medication was part of the exclusion criteria
Gorska-Ciebiada [26]	80.4% Oral agents, 47.1% insulin	N/A
Ascher-Svanum H [27]	89.9% Insulin	N/A
Zhang Y [11]	16% Insulin, 90.3% oral agents.	8.3% of the patients used psychotropic drugs
Zhang Y [11]	16% Insulin, 90.3% oral agents.	8.3% of the patients used psychotropic drugs
Luca A [29]	9.37% Diet, 54.6% oral agents, 35.9% insulin	N/A
Palta P [30]	19.6% Insulin, 80.4% oral agents	This was cited as a limitation
Zhang Y [28]	70% Oral agents, 30% insulin	7.3% of the patients used psychotropic drugs
Hayashino Y [31]	14.6% Diet, 45.5% oral agents, 39.9% insulin	N/A
Gorska-Ciebiada [32]	47.1% Insulin, 80.4% oral agents	N/A
Tsujii S [33]	15% No medication, 43.4% oral agents, 41.6% insulin	N/A
Mathew CS [34]	N/A	Use of depression medication was part of the exclusion criteria
Hamer M [35]	N/A	N/A
Stanković Z [36]	63% Insulin	The patients with repeated episodes of depression had not been on antidepressant treatment for at least one year before the inclusion or they were at the very beginning of the treatment with antidepressants.
Fisher L [37]	N/A	Use of psychotropic medication but not specified
Calhoun D [38]	19.3% Lifestyle, 51.6% oral agents, 14.3% insulin, 14.8% oral agents + insulin	N/A
Yu R [39]	18.5% Oral agents, 39.1% insulin	N/A
Egede LE [4]	42.3% Insulin	N/A
Lee HJ [40]	26.18% Oral agents, 36.4% insulin	N/A
Richardson LK [41]	19.15% Insulin	N/A
Daly EJ [42]	N/A	Use of depression medication was part of the exclusion criteria
de Groot M [43]	N/A	N/A

## Data Availability

Not applicable.
